# Phytol and bilimbi phytocompounds induce thermogenic adipocyte differentiation: An in vitro study on potential anti-obesity effects

**DOI:** 10.1016/j.heliyon.2024.e40518

**Published:** 2024-11-22

**Authors:** Farah Md Fauzi, Mohamad Faiz Hamzah, Muhd Zulkarnain Mahmud, Azimah Amanah, Mohd Hasnan Mohd Noor, Zafarina Zainuddin, Wai Kwan Lau

**Affiliations:** a Malaysian Institute of Pharmaceuticals & Nutraceuticals, National Institutes of Biotechnology Malaysia, Block 5A, Halaman Bukit Gambir, 11700, Gelugor, Penang, Malaysia; b Analytical Biochemistry Research Centre, Universiti Sains Malaysia, 11800, USM, Penang, Malaysia

**Keywords:** Obesity, Thermogenic adipocytes, Brown adipocytes, White adipocytes, *Averrhoa bilimbi*, Phytol, Squalene, *Ucp1*, *Prdm16*

## Abstract

Obesity is a major health concern associated to diabetes, cardiovascular disease, and cancer. Brown adipocytes, which specialise in thermogenesis, offer a potential therapeutic target for obesity prevention and related conditions. This study builds on previous findings of the browning activity of Averrhoa bilimbi hexane fractions and aims to elucidate the underlying mechanisms in vitro. Squalene and phytol, key phytocompounds from bilimbi leaf extract and fractions, were assessed for their ability to induce thermogenic adipocyte using 3T3-L1 preadipocytes and C2C12 myoblasts in vitro models. The result shows that bilimbi fractions F7, F8, and F9, along with squalene and phytol, effectively induced thermogenic adipocyte differentiation. This was evidenced by the upregulation of key markers, including *Ucp1*, *Prdm16*, and *Pgc1α*, and increased expression of the brown adipocyte-specific protein CIDEA in treated 3T3-L1 preadipocytes. Notably, all treatments promoted thermogenic adipocytes differentiation in C2C12 myoblasts via the upregulation of *Pgc1α, Ucp1* genes, and UCP1 protein. These findings suggest that bilimbi fractions and its phytocompounds may hold potential as nutraceutical interventions for obesity management.

## 1 Introduction

Obesity has become a global health crisis, characterised by excessive fat accumulation that significantly increases the risks of metabolic disorders, including type 2 diabetes, cardiovascular diseases, and certain cancers [1]. The growing prevalence of obesity highlights the urgent need for effective prevention and management strategies. Adipose tissues play a crucial role in regulating energy balance and metabolism, with three distinct types of adipocytes: white, brown, and beige [2].

White adipose tissue (WAT), the most abundant form of adipose tissue, stores energy as triglycerides and is closely associated with obesity and insulin resistance [3]. In contrast, brown adipose tissue (BAT), also known as thermogenic adipocytes, is involved in energy expenditure through non-shivering thermogenesis, helping to maintain body temperature and potentially reduce obesity by generating heat. This thermogenic activity is largely driven by the expression of the mitochondrial uncoupling protein-1 (*Ucp1*), which plays a central role in adaptive thermogenesis [4].

In addition to brown adipocytes, white adipocytes can undergo ‘browning’ under certain stimuli such as cold exposure and beta-adrenergic signalling, leading to the formation of beige adipocytes [5]. Like brown adipocytes, beige adipocytes are thermogenic, possessing mitochondria and expressing *Ucp1,* thereby contributing to increased energy expenditure [6]. However, beige adipocytes primarily arise from Myf5-negative precursor cells, while brown adipocytes originate from Myf5-positive precursors. Increased numbers of brown and beige adipocytes are associated with reduced adiposity, suggesting their therapeutic potential in obesity management [[7], [8], [9]].

One of the main challenges in utilising brown adipocytes for obesity management is their relatively low abundance in adults, especially in those with obesity [10]. This emphasises the need for interventions that can stimulate their formation and activity. Due to the side effects of synthetic anti-obesity drugs, there has been increasing interest in identifying safe, natural alternatives. Various plants, including chilli peppers, ginseng, cinnamon, green tea, raspberry, and capsaicin, have been reported to stimulate brown adipocyte differentiation and enhance energy expenditure [[11], [12], [13], [14], [15], [16]].

*Averrhoa bilimbi*, locally known as Belimbing Buluh, has demonstrated brown adipogenic effect like rosiglitazone, *Ppar-γ* agonist [17,18]. This effect is primarily mediated by the upregulation of PR domain-containing 16 (*Prdm16*) and *Ucp1*, which are key regulators of brown adipocyte development and function. Further studies have shown that specific hexane fractions of bilimbi are more effective in inducing browning than the crude extract and other extraction solvents [18].

The present study aims to identify the natural compounds present in these hexane fractions and elucidate their roles in thermogenic adipocyte differentiation at both transcriptional and translational levels. Besides, the thermogenic adipocyte differentiation potentials of two major phytocompounds in the bilimbi hexane fractions, namely squalene and phytol, are assessed.

## 2 Materials and methods

### 2.1 Bilimbi preparation and extraction

Bilimbi leaves were collected from an orchard with permission, and their authenticity was verified by a botanist with voucher specimen (No. 11,738) deposited in the herbarium of Universiti Sains Malaysia, Penang. The leaves were washed, dried, ground into powder, and subjected to extraction using hexane in a 1:10 ratio. The solvent was removed under reduced pressure at 40 °C using a rotary evaporator. Bilimbi fractions were then separated using optimized flash column chromatography and High-Performance Thin Layer Chromatography (HPTLC), employing silica gel plates as the stationary phase and a mobile phase of hexane and ethyl acetate (80:20). Phytol (97 %, a mixture of isomers) and squalene (≥98 %) were sourced commercially from Sigma (USA).

### 2.2 Fractionation process

The hexane bilimbi extract was fractionated into 10 distinct fractions. Each fraction was evaluated for its ability to induce brown adipocyte differentiation in C2C12 myoblasts. Fractions F7, F8, and F9, which showed the highest brown adipocyte differentiation percentages, were selected for further analysis (Fig. S1, Supplementary Files).

### 2.3 Characterisation of phytocompounds in extract and fractions

Phytocompound characterisations were performed using an Agilent 5977A Series Gas Chromatography-Mass Spectrometry (GC-MS) system. A 30 m × 0.25 mm x 0.25 μm ultra-inert column was used with electron ionisation (70 eV) as the detection method. Helium (99.9 %) served as the carrier gas at a flow rate of 1 mL/min. The GC oven temperature was ramped from 110 °C to 250 °C at 10 °C/min. The mass spectrometer scanned a range from 40 to 550 amu, with a split ratio of 1:25. Standard solutions of phytol, and squalene (1000 ppm) were dissolved in hexane. A 3 μL injection of bilimbi hexane extract and fractions (10 mg/mL) were analysed, and compounds were identified by comparing mass spectra with the Mass Hunter Library WION 14. L.

### 2.4 Cell culture and treatment

3T3-L1 preadipocytes (RRID: CVCL 0123) and C2C12 myoblasts (RRID: CVCL 0188) were obtained from the American Type Culture Collection (ATCC, USA) and authenticated using short tandem repeat (STR) analysis upon receipt [19]. Both cell lines were routinely tested for mycoplasma contamination and confirmed negative. Cells were maintained in Dulbecco's Modified Eagle's Medium (DMEM) supplemented with 10 % fetal bovine serum (FBS) in T-25 culture flasks.

For 3T3-L1 preadipocytes, differentiation was fully established before treatment to ensure the cells transitioned into mature adipocytes. Preadipocytes were seeded in 6-well plates at a density of 0.3 × 10^6^ cells per well. Once reaching confluency, differentiation was initiated using a hormonal differentiation cocktail of 0.5 mM isobutyl methylxanthine (IBMX), 5 μM dexamethasone (DEX), and 10 μg/mL insulin in DMEM with 10 % FBS [17]. After 2 days, the medium was replaced with DMEM containing 10 % FBS and 10 μg/mL insulin for an additional 4 days to complete the differentiation process. Following differentiation, bilimbi fractions (F7, F8, and F9) were added at 50 μg/mL. Squalene and phytol were administered at a concentration of 100 μM, following previously reported protocols [20,21]. Rosiglitazone (1 μM) and DMSO (0.1 %) were used as positive and negative controls, respectively. Treatments were replenished every 2 days throughout the 7-day treatment period.

For C2C12 myoblasts, differentiation and treatment were performed simultaneously [17]. After confluency, myoblasts were treated with 0.5 mM IBMX, 5 μM DEX, and 10 μg/mL insulin and bilimbi fractions, squalene, or phytol for 2 days. The medium was then replaced with DMEM containing 10 % FBS and 10 μg/mL insulin, along with the respective treatments, for another 2 days. The treatments were replenished every two days until oil droplet formation was observed. Rosiglitazone (1 μM) and 0.1 % DMSO were used as positive and negative controls, respectively. All cells were cultured in a 5 % CO_2_ incubator at 37 °C, with the medium replenished every 2 days throughout the experiment.

### 2.5 MTT assay

To assess cytotoxicity, 3T3-L1 preadipocytes, C2C12 myoblasts, and L929 fibroblasts were seeded at 1 × 10^5^ cells/well in 96-well plates and incubated at 37 °C under a 5 % CO_2_ atmosphere until confluence. Notably, 3T3-L1 preadipocytes and C2C12 myoblasts were cultured in DMEM supplemented with 10 % FBS, as described in Section 2.4, whereas L929 fibroblasts were cultured in MEM supplemented with 10 % FBS. Bilimbi fractions were prepared in serial two-fold dilutions (7.8125 μg/mL to 250 μg/mL), with 0.1 % DMSO as the blank control and sodium dodecyl sulfate (SDS) as the cytotoxicity control, which had a similar concentration range of 7.8125 μg/mL to 250 μg/mL. Cells were treated in triplicates for 7 days, with treatments replenished every 2 days. SDS was administered at day 6, a day before the MTT assay MTT reagent (1 mg/mL) was prepared in serum- and phenol-free DMEM or MEM, respectively. Subsequently, 50 μL of this solution was added to each well and incubated for 2 h at 37 °C. Isopropanol was introduced to dissolve the purple formazan crystals, and the plate was placed in a shaker for 30 min in the dark. Absorbance was read at 570 nm (with a reference wavelength of 650 nm) using a BMG Labtech microplate reader. Cell viability was calculated as a percentage of the control using the formula:

$$\text{Percentage}\;\text{of}\;\text{cell}\;\text{viability}=\left(100\times {OD}_{570\;\left(treatment\right)}\right)/{OD}_{570\left(blank\right)}$$

### 2.6 Oil Red O staining and quantification

C2C12 myoblasts were seeded into a 96-well plate at 1 × 10^5^ cells/mL and treated as described in Section 2.4. Following the differentiation and treatment period, cells were fixed with 4 % paraformaldehyde (PFA) for 1 h and washed with 1X PBS. Lipid droplets were stained with Oil Red O, prepared by dissolving 0.25g of the dye in 50 mL isopropanol. The solution was filtered and diluted in distilled water in 3:2 ratio before use. Cells stained for 1 h and then washed with 1X PBS. Morphological changes and intracellular lipid droplets were observed under an inverted phase-contrast microscope and the images were captured using Nikon. For quantification, Oil Red O was extracted by 100 % isopropanol and absorbance was measured at 570 nm using BMG Labtech microplate reader. 100 % isopropanol was used to control the background signal. The percentage of cell viability was calculated using the formula:

$$\text{Percentage}\;\text{of}\;\text{cell}\;\text{viability}=\left(100\times {OD}_{570\;\left(treatment\right)}\right)/{OD}_{570\;\left(blank\right)}$$

### 2.7 RT- PCR analysis

RNA was extracted using TRIzol Reagent (Ambion, Life Technologies) according to the manufacturer's protocol and quantified using a Nanodrop spectrophotometer. Gene-specific primers were designed using NCBI web-based BLAST tools and analysed using NetPrimer software (PREMIER Biosoft International, Palo Alto, CA), respectively. Quantitative real-time PCR (qRT-PCR) analysis was performed using the CFX96 Touch Real-Time PCR Detection System with Nexpro One-Step RT-qPCR Mastermix (NEX Diagnostics, Korea). Primers synthesised by Bioneer Corporation, Korea, were used at a concentration of 0.5 μM for each targeted gene. The primer sequences are provided in Table S1 in Supplementary Files.

The relative gene expression levels were determined using the Livak method's 2^-ΔΔCq^ [22]. Cq values were obtained from the expression of target genes in treated samples and normalizing it to the β-Actin reference gene. The ΔΔCq values were computed by comparing the ΔCq of the target gene to that of the control. The expression of bilimbi fractions was normalised, with DMSO-treated cells receiving a value of 1, representing a fold increase in expression.

### 2.8 Immunofluorescence assays

3T3-L1 preadipocytes were cultured and treated in a 96-well black tissue culture plate (PerkinElmer, USA). Cells were fixed with 4 % paraformaldehyde (PFA), permeabilised with Triton X-100, and blocked with 1 % bovine serum albumin (BSA) in PBS-Tween. Primary antibodies against DAPI (62248, Thermo Fisher, USA), rat P2X5 monoclonal antibody (sc-398682, Santa Cruz Biotechnology, USA), mouse PAT2 monoclonal antibody (sc-390969, Santa Cruz Biotechnology, USA), rabbit CIDEA polyclonal antibody (PA5-19908, Thermo Fisher, USA), rabbit ASC-1 polyclonal antibody (PA5-101174, Thermo Fisher, USA), and mouse β-actin monoclonal antibody (MA5-15739, Thermo Fisher, USA) were diluted in 1 % BSA in PBS-T at a 1: 500 dilution and incubated overnight at 4 °C. Fluorescent dye-conjugated secondary antibodies were added and incubated for 1 h at 37 °C. The secondary antibodies, including goat anti-mouse IgG (FITC)-conjugated (F-2761, Thermo Fisher, USA), goat anti-rabbit IgG (Texas-Red)-conjugated (T2767, Thermo Fisher, USA) and goat anti-at (FITC)-conjugated (31629, Thermo Fisher, USA), were used at a 1:500 dilution. Fluorescence signals were captured and quantified using a Fluorescence Imaging System and NIS-Element software. Protein expression was normalised to DAPI staining. The data were presented as a fold increase in expression relative to DMSO-treated cells, with a value of 1.

### 2.9 Prdm16 gene silencing

siRNA targeting of *Prdm16* (SI01387575, FlexiTube siRNA) and Hi-Perfect Transfection Reagent were used for transfection of *Prdm16* in C2C12 myoblasts. Positive and negative controls, named AllStars Cell Death Control siRNA (SI04939025) and AllStars Negative Control siRNA (1027280), respectively, were used. All siRNA reagents were purchased from Qiagen, Denmark. The siRNA concentration was 10 nM, and 3 μL of transfection reagent was applied per well in a 24-well plate, each well containing 3 × 10^4^ cells. The siRNA-transfection complexes were prepared and introduced onto C2C12 myoblasts. Following 48 h of transfection, the C2C12 myoblasts were treated as described in Section 2.4. Total RNA was extracted as described in Section 2.7. The *Prdm16* gene expression was determined by comparing 2^-ΔΔCq^ values normalised to β-actin. ΔΔCq compares the ΔCq values of the treated cells to the DMSO-treated group. The knockdown efficiency was calculated using the formula: 1–2 ^(-ΔΔCq^) x 100 %.

### 2.10 Western blotting analysis

C2C12 myoblasts were treated as described in Section 2.4. Protein lysates were extracted using Protein Extraction Reagent (Thermo Scientific, USA) supplemented with a protease inhibitor. Total protein concentrations were quantified using the Pierce BCA Protein Assay Kit, following the manufacturer's protocol, with bovine serum albumin (BSA) (500 μg/mL) serving as the standard. For each sample, 40 μg of protein was denatured and separated by SDS-PAGE at 100V for 2 h. The proteins were then transferred onto polyvinylidene difluoride (PVDF) membranes. To block non-specific binding, the membranes were incubated in 5 % BSA for 1 h at room temperature with gentle shaking. Following blocking, the membranes were incubated overnight at 4 °C with the following primary antibodies, diluted in 5 % BSA: UCP1 (D9D6X) rabbit monoclonal antibody #14670 (1:1000) and β-actin (13E5) rabbit monoclonal antibody #4970 (1:1000), both from Cell Signaling Technology. After primary antibody incubation, the membranes were washed with PBS-T containing 5 % BSA and then incubated with an HRP-cojugated anti-rabbit IgG (#7074; 1:2000; Cell Signaling Technology) for 1 h at room temperature. Immunoreactive bands were visualised using the Pierce ECL Western Blotting Substrate Solutions (Thermo Fisher, USA) and captured using a ChemiDoc XRS + Molecular Imager (Bio-Rad, USA). The band intensities were quantified using Image Lab™ Software version 5.2 (Bio-Rad, USA).

### 2.11 Statistical analysis

Data were presented as mean ± SEM of three independent experiments conducted in triplicate. Statistical analysis between treatment groups were analysed using one-way ANOVA followed by Dunnett's multiple comparison tests. A p-value of <0.05 was considered statistically significant.

## 3 Results

### 3.1 Fraction selection

The fractionation of the hexane extract from bilimbi leaves yielded 10 distinct fractions (Fig. S2, Supplementary Files), consistent with previous reports [18]. These fractions were screened for their ability to induce brown adipocyte differentiation in C2C12 myoblasts. Among them, F7, F8, and F9 demonstrated the most significant effects, inducing 70–80 % differentiation at a concentration of 50 μg/mL (data not shown). Due to their significant and consistent potency in driving brown adipocyte differentiation, F7, F8, and F9, were selected for further analysis.

### 3.2 Characterisation of phytocompounds

**Fig. 1:**
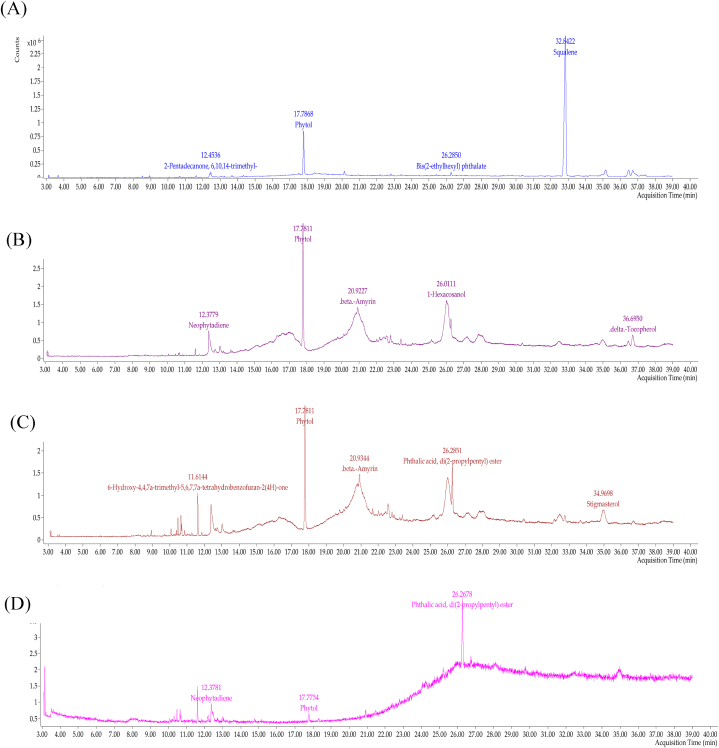
GC-MS chromatogram profiles showing (A) hexane bilimbi leaves extract, bilimbi fractions of (B) F7, (C), F8, and (D) F9.

The database search using the Mass Hunter Library WION 14. L revealed the main phytocompounds found in the extract and fractions of hexane bilimbi leav es (Table S2 and Table S3, Supplementary Files). The GC-MS chromatograms of phytol and squalene as standards are presented (Fig. S3, Supplementary Files). Squalene was significantly detected in hexane extracts at 96.9 % (Fig. 1A). Phytol was identified as the major phytocompound in bilimbi fractions F7 (Fig. 1B) and F8 (Fig. 1C), accounting for 98.5 % and 97.3 %, respectively. F9 was predominantly composed of phthalic acid (82.58 %) (Fig. 1D).

### 3.3 Cytotoxicity studies

**Fig. 2:**
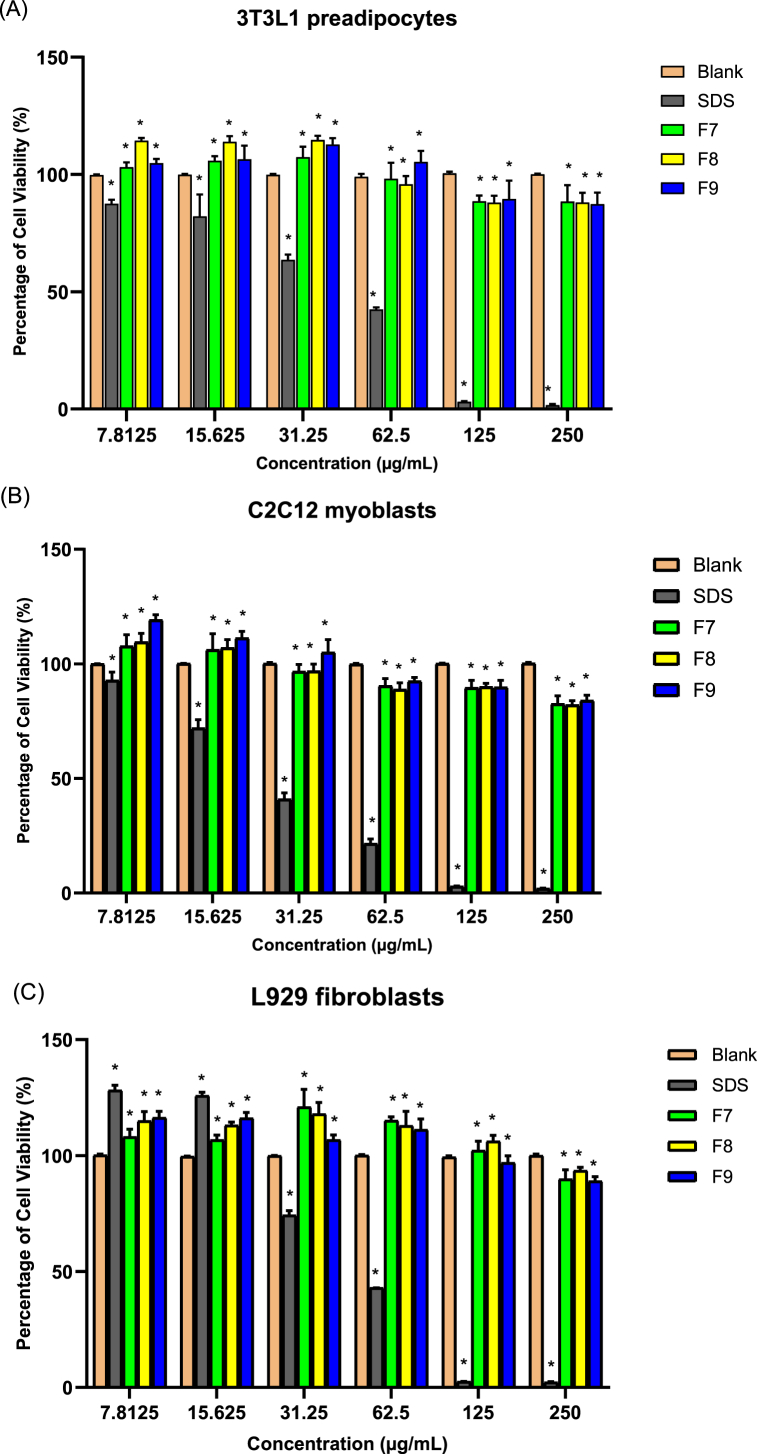
Percentage of cell viability in 3T3-L1 preadipocytes (A), C2C12 myoblasts (B), and L929 fibroblasts (C) after treatment with bilimbi fractions of F7, F8, and F9 at concentrations ranging from 7.8125 to 250 μg/mL for 7 days. SDS (sodium dodecyl sulfate) was used as a positive control for cytotoxicity. Data represents the mean ± SD of three independent experiments (n = 9), compared to untreated control samples. ∗ Indicates p < 0.05.

Cytotoxicity assays were performed on 3T3-L1 preadipocytes, C2C12 myoblasts, and L929 fibroblasts after 7 days of treatment with bilimbi fractions. None of the tested fractions (F7, F8, and F9) showed cytotoxicity at concentrations ranging from 7.8125 to 250 μg/mL, with more than 80 % cell viability observed even at the highest concentration (Fig. 2A, B, and 2C).

The IC_50_ values for each cell type ranged from 0.8 to 2.3 μg/mL, indicating low cytotoxicity across the tested concentrations. Notably, at the lowest concentrations of bilimbi fractions, an increase in cell viability was observed, which may suggest promotion of cell propagation or no adverse effects on cell growth at these concentrations. In contrast, SDS exhibited a cytotoxic effect, with a significant decrease in cell viability observed at 60 μg/mL in 3T3-L1 preadipocytes (Fig. 2A) and L929 fibroblasts (Fig. 2C), and at 30 μg/mL in C2C12 myoblasts (Fig. 2B), indicating a more pronounced toxicity relative to bilimbi fractions.

### 3.4 Brown adipogenic differentiation of C2C12 myoblasts

**Fig. 3:**
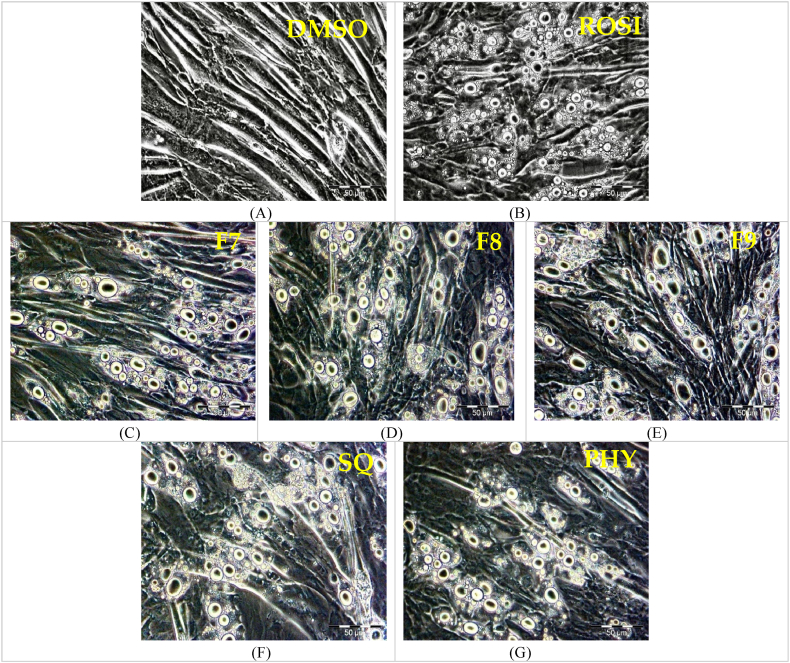
Differentiation of C2C12 myoblasts with lipid droplet formation. (A) negative control treated with 0.1 % DMSO, (B) positive control treated with 1 μM rosiglitazone. (C), (D) and (E) represent C2C12 myoblasts treated with bilimbi fractions F7, F8, and F9 at 50 μg/mL, respectively. (F) and (G) show myoblasts treated with phytol and squalene at 100 μM, respectively. The cells were incubated in each treatment for 7 days, and images were captured under x20 magnification. Scale bar = 50 μM.

The formation of lipid droplets is a well-established marker of brown adipocyte differentiation, as previously reported [23]. In this study, untreated C2C12 myoblasts in the presence of 0.1 % DMSO were observed to form myotubes after confluent (Fig. 3A). Notably, rosiglitazone, a *Pparγ* agonist, has been shown to promote the differentiation of C2C12 myoblasts into brown adipocytes [24]. Consistent with this, rosiglitazone-treated cells in this study also exhibited a significant accumulation of lipid droplets (Fig. 3B). This effect was similarly observed in cells treated with bilimbi fractions F7 (Fig. 3C), F8 (Fig. 3D), F9 (Fig. 3E), as well as in those treated with squalene (Fig. 3F) and phytol (Fig. 3G).

While these findings suggest a strong potential for these compounds to induce brown adipocyte differentiation, further molecular studies are necessary to confirm the observed phenotypic changes at the gene and protein levels.

### 3.5 Effect of Prdm16 silencing on Brown adipocyte differentiation

**Fig. 4:**
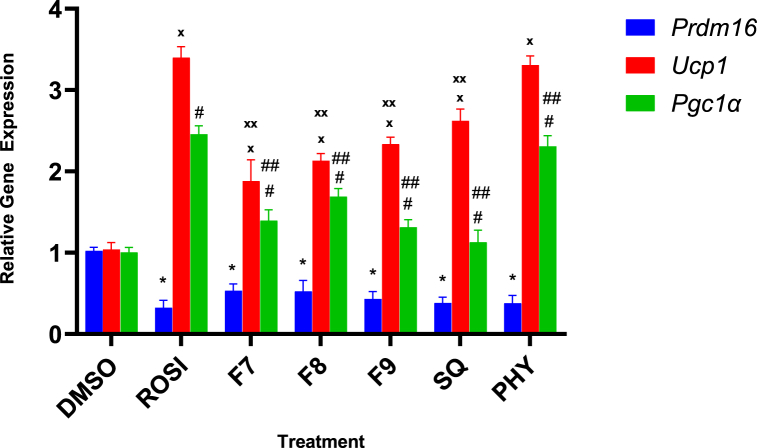
Effect of bilimbi fractions and phytocompounds on the mRNA expression level of *Prdm16*, *Pgc1α*, and *Ucp1* in C2C12 myoblasts transfected with siRNA *Prdm16*. The negative control was treated with 0.1 % DMSO (v/v), and the positive control with 1 μM rosiglitazone (ROSI). Bilimbi fractions (F7, F8, F9), were administered at 50 μg/mL, and squalene (SQ) and phytol (PHY) at 100 μM. Results were normalised to β-actin and expressed as relative mRNA expression levels compared to non-treated cells. Statistical significance in mRNA expression was determined by one-way ANOVA followed by multiple comparisons using Dunnett's post hoc test, with p ≤ 0.05 considered statistically significant (n = 9). Symbols: (∗), (**ₓ**), (#), represent significant differences compared to DMSO-treated cells in *Prdm16*, *Pgc1α*, and *Ucp1*, respectively. Symbols: (∗∗), (**ₓₓ**), (##), represent significant differences compared to ROSI-treated cells in *Prdm16*, *Pgc1α*, and *Ucp1*, respectively.

In the presence of *Prdm16*, C2C12 myoblasts preferentially develop to brown adipocytes rather than myotubes [25,26]. To further investigate if *Prdm16* serves as the regulatory target in brown adipocyte induction, C2C12 myoblasts with silenced *Prdm16* were treated with bilimbi fractions, squalene, and phytol (Fig. 4).

**Fig. 5:**
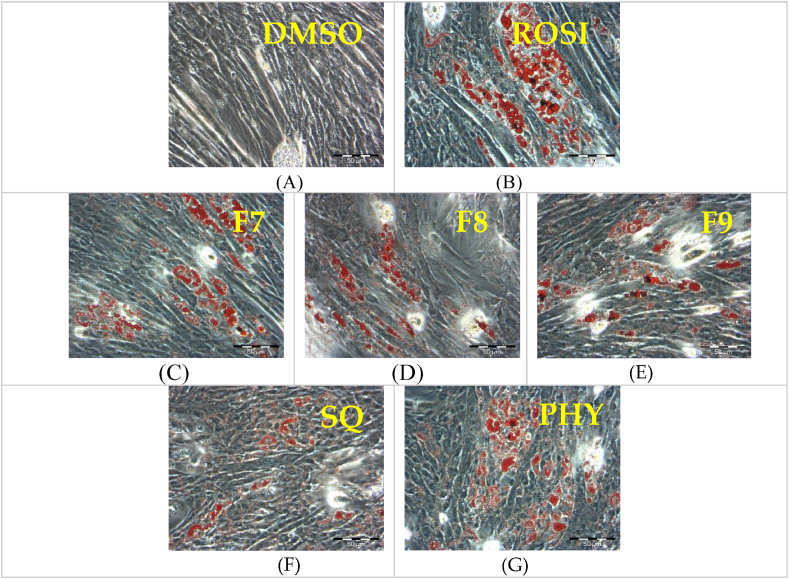
Representative images of Oil Red O staining of *Prdm16*-silenced C2C12 myoblasts with treatments. Transfection with siRNA-*Prdm16* was performed for 48 h, followed by a 7-day treatment period. The negative control received 0.1 % DMSO (v/v) (A) and the positive control was treated with 1 μM rosiglitazone (ROSI) (B). Bilimbi fractions of F7 (C), F8 (D), F9 (E) were administered at 50 μg/mL, and squalene, SQ (G) and phytol, PHY (F) at 100 μM. Oil-red O staining was performed on day 8 to assess lipid droplet formation, with images captured at x20 magnification, and a scale bar representing 50 μM.

The transfection efficiency of siRNA *Prdm16* on C2C12 myoblasts was validated via qRT-PCR against different siRNA controls. *Prdm16* was successfully silenced achieving knockdown efficiency of 70–90 % (Fig. S4, Supplementary Files). Surprisingly, *Prdm16* knockdown did not directly affect the expression of the *Pgc1α* and *Ucp1* genes. Treatments significantly upregulated these genes, with rosiglitazone and phytol being the most effective, leading to a remarkable 3.5-fold increase in *Ucp1* expression and a >2.0-fold increase in *Pgc1α* expression, surpassing other treatments (Fig. 4). Treatment with F7 induced *Ucp1* and *Pgc1α* by 2-fold and 1.5-fold, respectively. F8 treatment increased the expression of *Ucp1* and *Pgc1α* by more than 2.5-fold and 2-fold times, respectively, almost comparable to the effects of F9 and squalene. Notably, *Ucp1* consistently exhibited higher expression than *Pgc1α* across all treatments following transfection and treatment administration. Silencing of *Prdm16* had no effect on brown adipogenesis and myogenesis in the presence of rosiglitazone, bilimbi fractions, phytol, and squalene, as evidenced by Oil Red O staining of lipid droplet accumulation (Fig. 5).

**Fig. 6:**
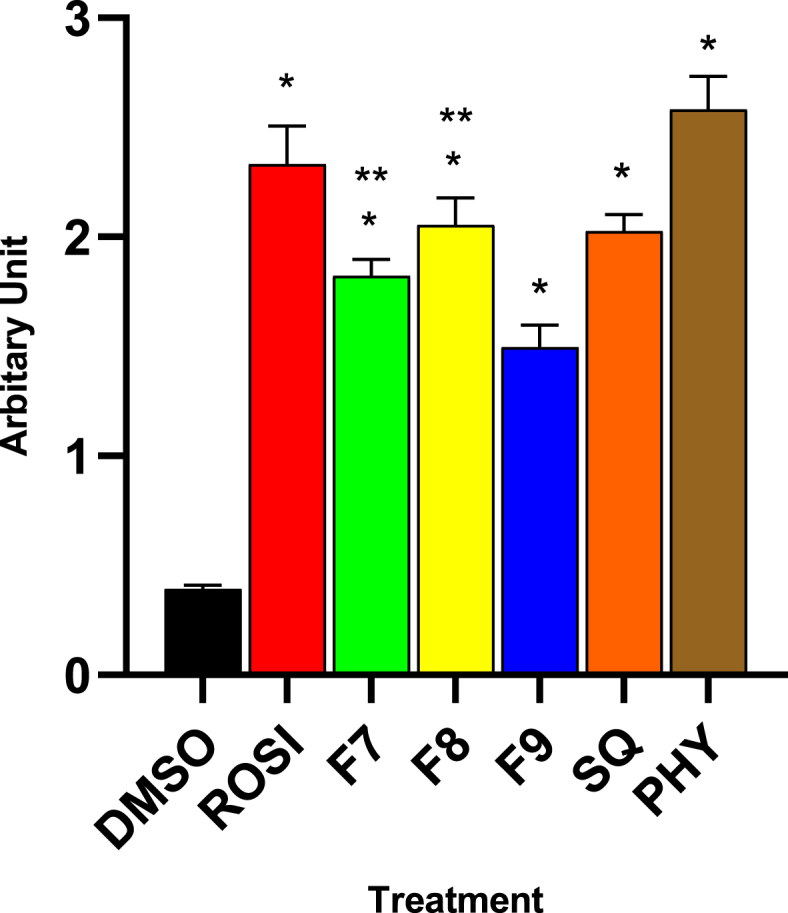
Quantitative analysis of Oil Red O staining was used to evaluate lipid accumulation following treatments. Statistical significance compared to DMSO is denoted as (∗), and statistical significance compared to ROSI-treated cells is denoted as (∗∗). Statistical significance was determined by one-way ANOVA followed by Dunnett's post hoc test, with p ≤ 0.05 considered statistically significant (n = 9).

As expected, no adipogenesis occurred, and the C2C12 myoblasts matured into myotubes in non-treated *Prdm16*-silenced cells. This was confirmed by the characteristic morphology of elongated and multinucleated myotubes (Fig. 5A). In contrast, the treated *Prdm16-*silenced C2C12 myoblasts exhibited the presence of lipid droplets, as indicated by Oil Red O staining (Fig. 5B–G). The red-stained lipid droplets visible within the cells mark an adipogenic shift, demonstrating that treatment can overcome the *Prdm16* silencing effect, enabling adipocyte differentiation. In addition, a quantitative analysis was performed to further validate the adipogenic shift in the treated *Prdm16-*silenced C2C12 myoblasts (Fig. 6).

While Oil Red O staining (Fig. 6) demonstrated a 2.5-fold increase in lipid droplet accumulation with rosiglitazone and phytol, along with a substantial increase with other treatments, this method primarily indicates lipid accumulation, which is a hallmark of adipogenesis. However, it does not provide direct evidence of changes in protein expression involved in thermogenic adipocyte differentiation. Therefore, additional analysis, such as Western blot, are necessary to evaluate the activation of key thermogenic proteins, such as UCP1 protein, to confirm the differentiation and thermogenic potential of brown adipocytes.

### 3.6 UCP1 thermogenic properties

**Fig. 7:**
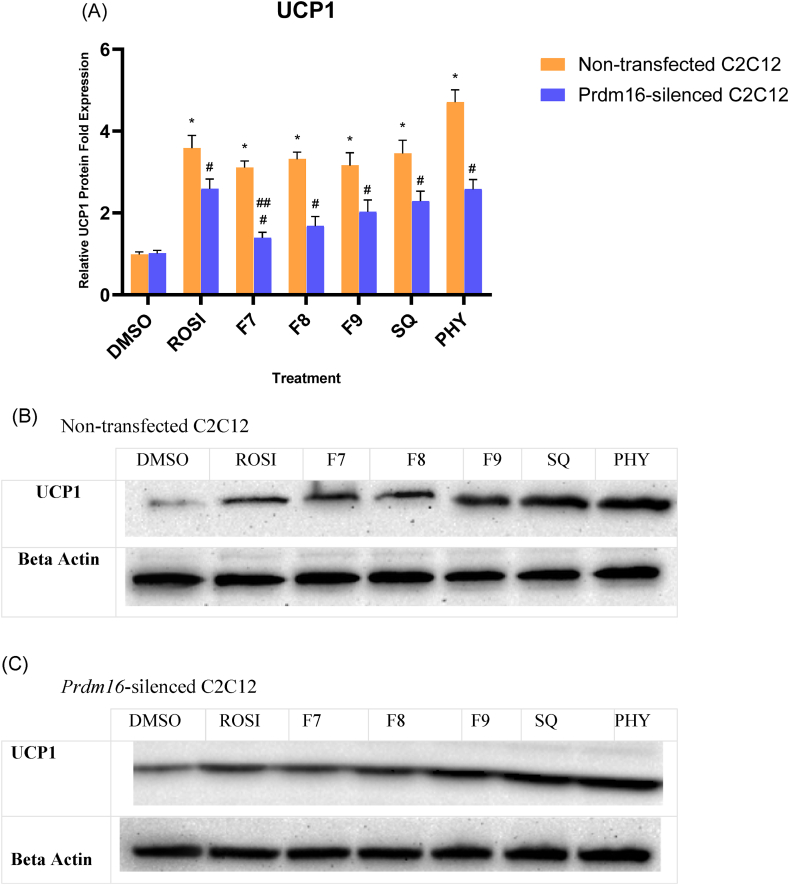
Western Blot analysis of UCP1 protein levels in non-transfected and siRNA-*Prdm16* silenced C2C12 myoblasts after respective treatments. The negative control received 0.1 % DMSO (v/v) and the positive control was treated with 1 μM Rosiglitazone (ROSI). Bilimbi fractions (F7, F8, F9) were administered at 50 μg/mL, and squalene (SQ) and phytol (PHY) at 100 μM. (A) Quantification of UCP1 protein band intensities, normalised to β-actin, showing relative UCP1 expression levels compared to the DMSO control. (∗) indicates statistically significance (ANOVA P < 0.05) compared to the DMSO (vehicle) and (∗∗) denotes statistically significance (ANOVA P < 0.05) compared to ROSI (positive control). (B) Representative Western blot image of the UCP1 and β-actin protein in non-transfected C2C12 myoblasts. (C) Representative Western blot image of UCP1 and β-actin protein in siRNA-*Prdm16* silenced C2C12 myoblasts. β-actin was used as the loading control in all experiments. Band intensities were quantified using Image Lab™ Software version 5.2 (Bio-Rad, USA).

To further substantiate the effect of *Prdm16* silencing on brown adipocyte differentiation and subsequent cellular thermogenesis, a Western Blot analysis was performed to evaluate the activation of UCP1 upon knockout of *Prdm16* in C2C12 myoblasts. All the protein data was normalised to β-actin as the loading control (Table S4, Supplementary Files). In the presence of *Prdm16*, UCP1 protein expression was markedly upregulated in response to all treatments, with the highest levels observed in phytol-treated cells, as shown in Fig. 7A, and further supported by the representative blots in Fig. 7B (Figure S5-Figure S6, Supplementary Files). Notably, UCP1 levels in cells treated with bilimbi fractions were comparable to those observed with rosiglitazone, highlighting their potential efficacy in promoting thermogenesis.

Interestingly, in siRNA *Prdm16-*silenced C2C12 myoblasts, UCP1 expression remained upregulated despite the silencing of *Prdm16* (Fig. 7C, Figure S7-Figure S8, Supplementary Files), which suggests that *Prdm16-*independent pathways may also contribute to the induction of UCP1. These results reflect that while *Prdm16* plays a role in brown adipocyte differentiation, may not be the main regulatory target of thermogenesis activation. Treatments with bilimbi fractions, squalene, and phytol have been observed to potentially upregulate UCP1 on protein level*,* even in the absence of *Prdm16* highlights their potential to promote thermogenic adipogenesis via alternative pathways.

### 3.7 Expression of adipogenesis and Brown adipocyte-related genes

**Fig. 8:**
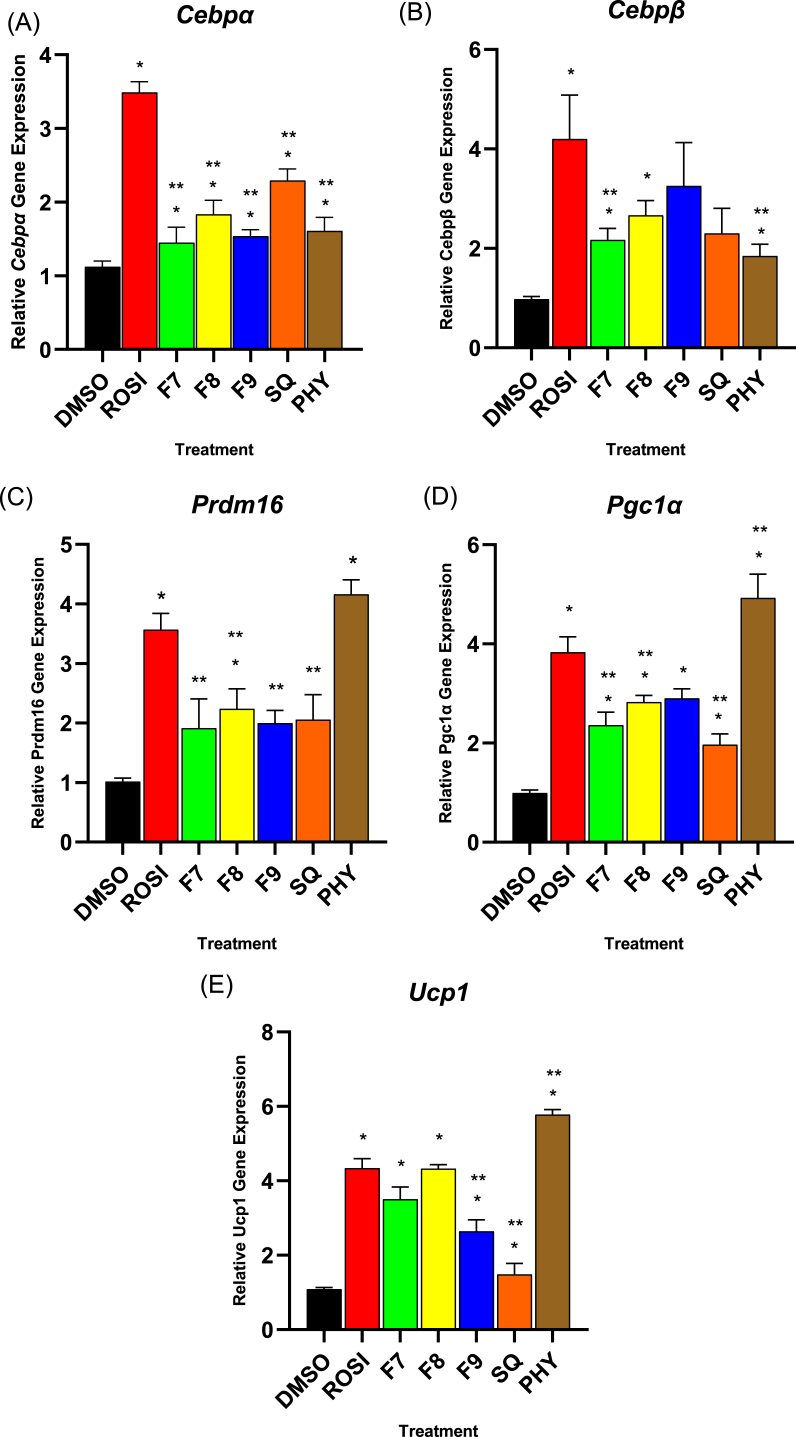
Effect of bilimbi fractions (F7, F8, F9), phytol, and squalene on mRNA expression of adipogenesis (*Cebpα* and *Cebpβ*) and mRNA brown adipocyte (*Prdm16*, *Pgc1α* and *Ucp1*)- in treated 3T3-L1 preadipocytes. (A) mRNA expression of *Cebpα*, (B) *Cebpβ*, (C) *Prdm16*, (D) *Pgc1α,* and (E) *Ucp1.* Treatments included 1 μM rosiglitazone (ROSI) as a positive control, 0.1 % (v/v) DMSO as the vehicle control, 50 μg/mL for bilimbi fractions (F7, F8, F9), and 100 μM phytol and squalene. mRNA expression levels, normalised to β-actin, are shown relative to DMSO control. Data represent the mean ± SD of three independent experiments (n = 9). Statistical significance (∗) was determined using one-way ANOVA followed by Dunnett's post hoc test, with p ≤ 0.05 considered statistically significant. Statistical significance between rosiglitazone and other treatments is indicated by (∗∗) at p ≤ 0.05.

In this study, 3T3-L1 preadipocytes, which typically representing ‘white’ adipocytes were treated with bilimbi fractions, squalene, and phytol to evaluate the effect of the treatments on white-to-beige differentiation. Key adipogenic markers such as *Cebpα* and *Cebpβ,* were measured alongside thermogenic markers like *Pgc1α, Ucp1*, and *Prdm16* to determine the shift towards a thermogenic, beige adipocyte phenotype (Fig. 8). The expression levels of these genes were normalised to the non-treated control cells, which were set to a baseline value of 1, allowing the comparison of the relative fold increase in gene expression across treatments.

With respect to adipogenesis, bilimbi fractions F7, F8, and F9 significantly upregulated the mRNA expression of two key adipogenesis-related marker genes of *Cebpα* and *Cebpβ* (Fig. 8A and B) by nearly 2-fold, respectively compared to the non-treated cells. By contrast, rosiglitazone, used as a positive control, resulted in a more pronounced effect, with nearly 3.5-fold increase in both *Cebpα* and *Cebpβ* expression, statistically outperformed the bilimbi fractions (p ≤ 0.05). This highlights rosiglitazone as an effective benchmark for adipogenesis induction. Squalene induced a 2.5-fold increase in *Cebpα* expression, while its effect on *Cebpβ* was comparable to the bilimbi fractions, showing a nearly 2-fold increase. Phytol treatment resulted in a 1.5-fold increase in the expression of both *Cebpα* and *Cebpβ.* Overall, *Cebpβ* expression was comparable across bilimbi fractions, squalene, and phytol, with increases ranging from 1.5 to 2.5-fold.

In terms of brown adipocyte differentiation, phytol significantly induced the expression of key thermogenic marker genes, including *Prdm16* (Fig. 8C), *Pgc1α* (Fig. 8D) and *Ucp1* (Fig. 8E), resulting in a remarkable 4–5.5-fold increase, which was statistically higher than the effect observed with rosiglitazone (p ≤ 0.05). Specifically, for *Prmd16* (Fig. 8C), rosiglitazone increased its expression by 3.5-fold, while F8 achieved a 2.5-fold increase, which is comparable to F7, F9, and squalene. For *Pgc1α* (Fig. 8D), rosiglitazone resulted in a >3.5-fold increase, and a similar 3-fold expression was observed across all bilimbi fractions, while squalene induced a modest 2-fold increase. Notably, *Ucp1* expression (Fig. 8E) was 4-fold higher in rosiglitazone and F8 than in the other brown-related genes. Importantly, phytol treatment significantly surpassed the expression of *Ucp1* relative to rosiglitazone (p ≤ 0.05), indicating its strong potential in promoting thermogenic adipocyte differentiation.

These results suggest that the bilimbi fractions, squalene, and phytol not only promote adipogenesis, but also drive the expression of thermogenic key genes, signifying the white-to-beige conversion, with phytol demonstrating the most potent effects.

### 3.8 Protein expression of adipocyte-specific protein markers

**Fig. 9:**
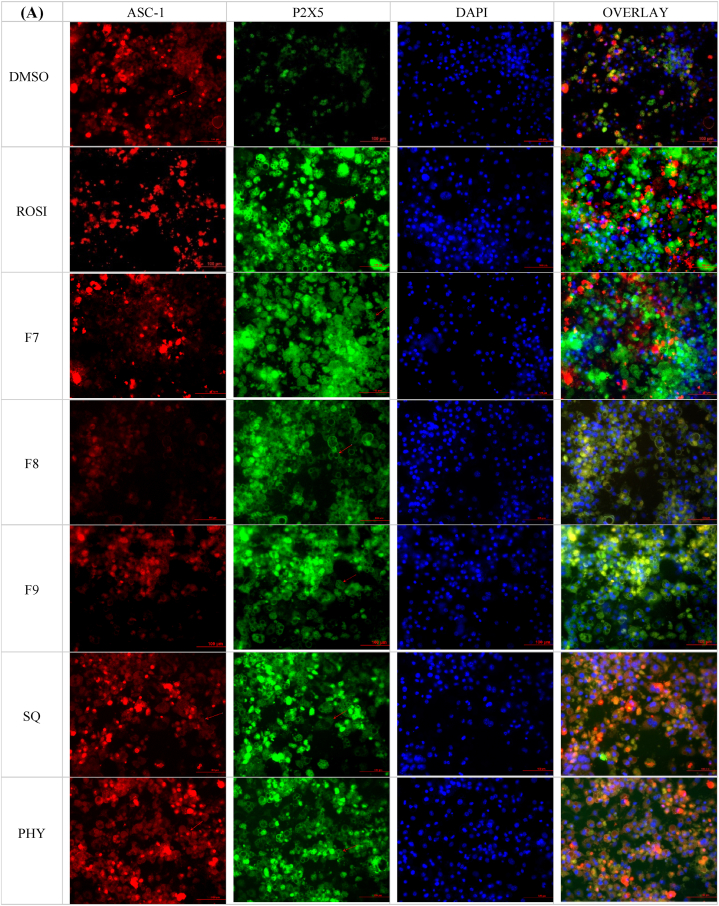

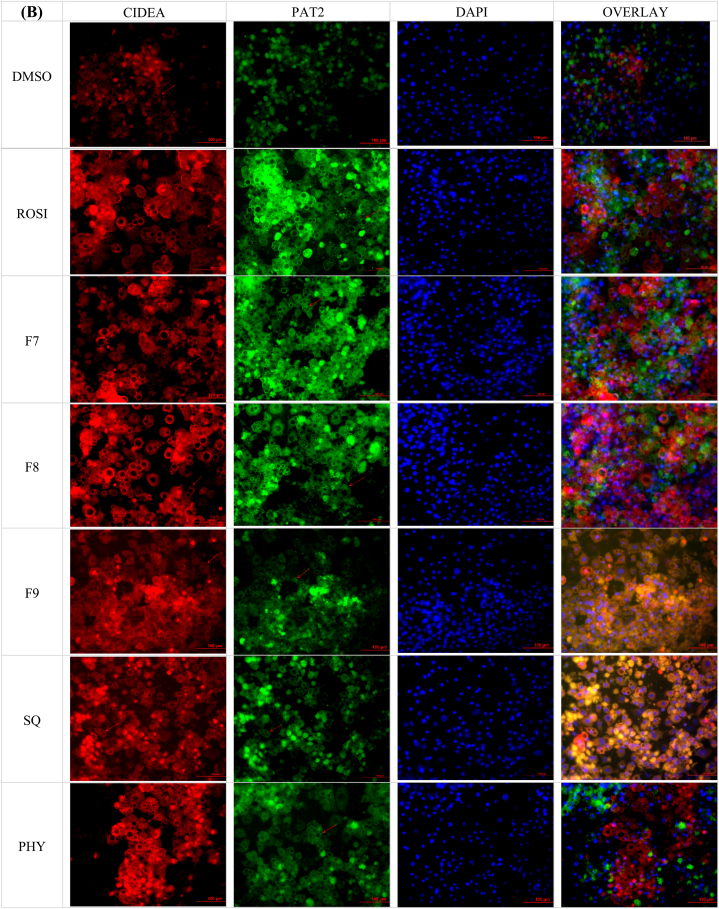
(A) Immunofluorescence microscopy of the treated 3T3-L1 preadipocytes. Red staining by Texas red represents ASC-1 and green staining by FITC represents P2X5. (B) Red staining by Texas red represents CIDEA and green staining by PAT2. Blue staining in A and B represents (DAPI). Red arrows indicate lipid droplets. The negative control was treated with 0.1 % DMSO (v/v), the positive control with 1 μM rosiglitazone (ROSI), bilimbi fractions (F7, F8, F9) at 50 μg/mL and both squalene (SQ) and phytol (PHY) were administered at 100 μM. The immunofluorescence images were captured at x20 magnification. Scale bar, 100 μM.

Following the assessment of gene expression, the investigation was extended to evaluate the translational effects of bilimbi fractions, squalene, and phytol on white-to-beige conversion at the protein level. Immunostaining of key adipocyte-specific protein markers, including CIDEA, P2X5, PAT2, and ASC1, was performed to assess their localisation and expression in treated cells. These markers, chosen for their established roles in adipocyte phenotypes [27], were assessed for their localisation and expression in treated 3T3-L1 adipocytes. Immunofluorescence microscopy demonstrated the localisation of these proteins around the lipid droplets as shown in Fig. 9, with captured intensities digitised and normalised to the DAPI-based cell count. Although P2X5, PAT2, and ASC-1 are surface markers, their visualisation near lipid droplets in Fig. 9 may reflect their involvement processes related to lipid metabolism.

**Fig. 10:**
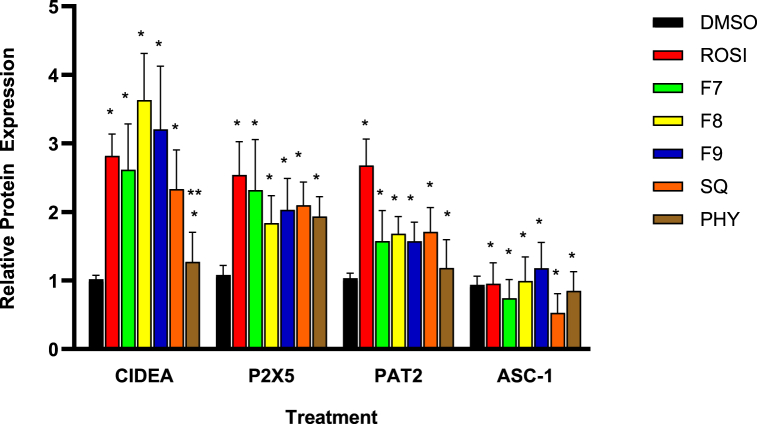
Effect of bilimbi fractions (F7, F8, F9), squalene, and phytol on the expression of adipocyte-specific protein markers (CIDEA, P2X5, PAT2, and ASC-1) in 3T3-L1 preadipocytes. The negative control was treated with 0.1 % DMSO (v/v), the positive control with 1 μM rosiglitazone (ROSI). Bilimbi fractions were administered at 50 μg/mL, and squalene (SQ) and phytol (PHY) at 100 μM. Results are presented as the mean ± SD of four independent experiments (n = 12), compared to non-treated controls. Statistical significance (∗) was determined using one-way ANOVA followed by multiple comparisons using Dunnett's post hoc test (p ≤ 0.05). (∗∗) indicates statistical significance. compared to rosiglitazone (ROSI).

ASC-1, a surface marker for white adipocytes, exhibited low-intensity red staining across all treatments (Fig. 9A), indicating a reduction in white adipocyte characteristics. This reduction suggests that treatments with bilimbi fractions, squalene, and phytol potentially suppress white adipocyte phenotype, favouring phenotypic conversion. In contrast, P2X5, a beige adipocytes marker, showed significant green-staining, particularly with bilimbi fraction F7, and substantial expression across other treatments. This supports the ability of the treatments to induce white-to-beige adipocyte conversion. CIDEA, a key thermogenic marker, demonstrated significant red staining across all treatments, including rosiglitazone, bilimbi fractions, phytol, and squalene (Fig. 9B). This suggests that the treatments enhance CIDEA protein expression and thermogenic activity. Similarly, PAT2, another beige adipocyte marker, exhibited a strong green staining across all treatments (Fig. 9B), further highlighting the enhancement of beige adipocyte differentiation and metabolic functionality. Quantification of relative protein expression, normalised to non-treated cells, confirmed the effects of these treatments (Fig. 10).

With respect to CIDEA, a marker specific to thermogenic adipocytes, treatments with F8 and F9 resulted in a substantial increase in expression, exceeding 3.5-fold compared to the DMSO control outperforming rosiglitazone, which induced a >2.5-fold increase (Fig. 10). F7 treatment led to a >2-fold increase, while squalene and phytol induced modest increases of 2-fold and >1.5 fold, respectively. Rosiglitazone was shown to enhance the expression of P2X5, a beige adipocyte-specific marker, by 2.5-fold, upregulation was also observed F8, F9, squalene, and phytol treatments with around 2fold increase. The expression of PAT2, another beige adipocyte marker, was also shown upregulated by more than a 2.5-fold in the presence of rosiglitazone. Similarly, all fractions, squalene, and phytol showed modest effects, with less than 2-fold increase. ASC-1, a marker for white adipocytes, exhibited lower expression across all treatments, with no significant increases observed. These findings indicate that the bilimbi fractions, squalene, and phytol not only modulate gene expression, but also influence the expression of key adipocyte-specific proteins, further supporting their roles in stimulating white-to-beige adipogenic conversion.

## 4 Discussion

In this study, the potential of bilimbi fractions, squalene, and phytol to stimulate brown and beige adipocyte differentiation was explored, building on the premise that brown and beige adipocytes represent promising therapeutic targets for addressing obesity and metabolic disorders. These cells possess unique thermogenic capabilities, which contribute to increased energy expenditure and fat utilisation. It has been well-documented that white adipocytes can adopt brown-like features in response to stimuli such as cold exposure or direct β-adrenergic stimulation [28,29]. Rosiglitazone, an established *Ppary* agonist, has also been shown to induce browning in adipocytes [24,30], making it a benchmark for comparison in this study. By evaluating bilimbi fractions, squalene, and phytol, this study aimed to identify alternative or complementary agents to rosiglitazone in stimulating thermogenic adipocyte differentiation.

The bioassay-guided fractionation of bilimbi hexane extracts yielded 10 distinct fractions, as characterised through chromatographic separation, with the fraction profiles matching prior reports [18]. Phytol, abundantly present in F7 and F8, and phthalic acid in F9, were identified as major phytocompounds. Phytol has been recognised for its role in stimulating brown adipocyte differentiation via the AMPK-α signalling pathway [20], while squalene has been reported to increase HDL cholesterol in vivo and promote adipogenesis in vitro [21,31]. These findings align with the current study's results, where bilimbi fractions, squalene, and phytol significantly upregulated brown adipocyte-related markers including *Ucp1*, *Pgc1α,* and *Prdm16.* Other identified phytocompounds such as δ-tocopherols, have demonstrated thermogenic effects by upregulating UCP1 protein and *Pgc1α* expressions through p38 MAPK activation, further supporting the potential of bilimbi fractions in stimulating browning activity [32]. Hexacosanol activates AMPK, inhibiting cholesterol biosynthesis and inducing hepatic lipogenic gene expression [33]. In contrast, stigmasterol suppresses hepatic lipogenic gene expression and reduces circulating ceramide levels, counteracting hyperlipidemia and obesity [34]. Additionally, 2(4H)-benzofuranone, 5,6,7,7a-tetrahydro-4,4,7a-trimethyl-, (R), has been effective in reducing hyperglycemia, dyslipidemia, and obesity in vivo [35]. β-amyrin inhibits preadipocyte differentiation and suppresses adipogenic transcription factors such as *Ppparγ* and *Cebpα* in vitro by activating the AMPK-α signalling pathway [36]. While the screening approach successfully identified multiple bioactive compounds, each has been previously reported to have thermogenic adipocyte differentiation through distinct mechanisms. Regulatory pathways of individual compounds should be further studied to better evaluate their therapeutic potential in obesity and related metabolic disorders.

Despite previous cytotoxic reports [18], this study demonstrated favourable cytotoxicity profiles of the plant fractions at concentrations up to 250 μg/mL, with promising cell viability observed across 3T3-L1 preadipocytes, C2C12 myoblasts, and L929 fibroblasts. The inferred IC_50_ values, which range between 0.8 and 2.3 mg/mL, further support the non-toxic nature of the bilimbi fractions. These findings are promising, particularly when considering the potential for safer alternatives to synthetic anti-obesity drugs, such as rosiglitazone, which has been associated with cardiovascular risks. Bilimbi fractions, squalene, and phytol exhibited comparable browning effects, suggesting their potential as natural and safer therapeutic options for obesity management. Further studies, however, are required to assess long-term efficacy and safety in in vivo models.

Two well-established in vitro models- C2C12 myoblasts and 3T3-L1 preadipocytes-were used to investigate the thermogenic adipocyte differentiation effects. C2C12 myoblasts, derived Myf5-positive progenitors, serve as a model for brown adipocyte due to their shared lineage with brown adipocytes [37]. Under specific conditions, these myoblasts can be induced to express brown adipocyte-specific genes, such as *Ucp1.* Meanwhile, 3T3-L1 preadipocytes were chosen for their ability to differentiate into white adipocytes, which, under appropriate stimuli, can undergo white-to-beige conversion [29]. This conversion is assessed by the expression of thermogenic adipocyte-specific markers following treatment with bilimbi fractions and phytocompounds. *Prdm16*, a well-established regulator of brown adipocyte differentiation, has been implicated in various extracts, including green tea, black raspberry, and bilimbi leaves [38]. Consistent with previous studies, our data confirmed the involvement of *Prdm16* in rosiglitazone-induced browning, where *Prdm16* expression was significantly upregulated. However, bilimbi fractions, squalene, and phytol-induced browning appeared to occur independently of *Prdm16,* as its knockdown did not hinder the upregulation of key brown adipocyte markers, such as *Ucp1* and *Pgc1α*. Interestingly, in siRNA*-Prdm16* transfected C2C12 myoblasts, where *Prdm16* expression is knocked down, the trend of increased UCP1 protein expression following treatment with bilimbi fractions, squalene, and phytol was still observed, albeit at lower levels than in non-transfected C2C12 myoblasts. This suggests that these compounds may activate *Ucp1* through *Prdm16*-independent pathways or partially compensate for the reduced *Prdm16* activity. This finding is particularly important in the context of metabolic disorders where *Prdm16* function may be compromised, indicating that these natural compounds could offer therapeutic benefits even under such conditions*.* While *Prdm16* is considered essential for various activities, such as brown adipogenesis, thermogenesis, and adipogenic differentiation of myoblasts, the results herein revealed the dispensability of *Prdm16* for the browning process induced by bilimbi fractions, phytocompounds, or rosiglitazone. This unexpected finding suggests the existence of alternative pathways for browning, potentially mediated by related genes such as *Ucp1* or other related-*Prdm* family (*Prdm3*) [39]. These findings open new avenues for research into the precise mechanisms through which natural compounds such as phytol stimulate thermogenesis.

Brown adipocyte differentiation begins with a commitment to adipogenesis, which includes the expression of early adipogenic genes such as CCAT/enhancer-binding protein *Cebpβ* and *Cebpδ*. This process is driven by the master regulator of adipogenesis, peroxisome proliferator-activated receptor-gamma (*Pparγ*) [40]. This study demonstrated that bilimbi fractions, phytol, and squalene upregulated *Cebpβ* and *Cebpα*, although to a lesser extent than rosiglitazone. Rosiglitazone treatment increased expression of *Cebpα*, which is known to have an antagonistic effect when silenced [41]. Interestingly, this study suggests that while rosiglitazone is known to stimulate *Cebpβ* expression, bilimbi fractions, particularly F8, also modestly induced this gene, pointing out their potential in promoting brown adipocyte differentiation through similar pathways.

PR domain containing 16 (*Prdm16*), a key transcriptional regulator in specifying brown adipocyte differentiation, interacts with *Cebpβ* to induce the expression of key brown adipocyte genes, such as the nuclear coactivator PPAR-γ coactivator 1α (*Pgc1α*) and uncoupling protein 1 (*Ucp1*) [42]. Previous studies have shown that *Prdm16* is essential for the browning activity induced by rosiglitazone [24]. Consistent with these findings, the current study observed a significant induction of *Prdm1*6 by rosiglitazone and a modest expression by bilimbi fractions, particularly in F8, indicating its potential role in browning activity. Notably, phytol exhibited significantly higher expression of all brown-related markers, exceeding that of rosiglitazone, which indicates its potent browning activity. The similar expression levels of *Pgc1α* and *Ucp1* observed between rosiglitazone and bilimbi fractions align with previous reports of increased metabolic rates associated with these treatments [17]. *Pgc1α*, a master regulator of brown adipocyte development, is critical for mitochondrial biogenesis and metabolism [43]. *Ucp1*, an uncoupling mitochondrial protein exclusive to brown adipocytes, plays a key role in heat dissipation and enhanced fat utilisation [44].

In terms of protein expression, the upregulation of CIDEA, P2X5, and PAT2—markers specific to brown and beige adipocytes—highlighted the potential of bilimbi fractions, squalene, and phytol in promoting the browning process. Notably, CIDEA, a key marker for brown adipocyte function, was significantly elevated in response to F8 and F9 treatments, surpassing the effects of rosiglitazone. This suggests that these fractions, in particular, may have potent browning effects. While ASC-1 may have been used as a white adipocyte marker in the study, its reduction in expression aligns with the browning process and supports the observed metabolic changes towards thermogenesis. A recent study also reported reduced ASC-1 expression following rosiglitazone treatment [45].

*Despite these promising findings, future studies should be carried out in animal models to assess the therapeutic potential of bilimbi fractions, squalene, and phytol in a more holistic biological context to address more complex physiological responses. Furthermore, signalling pathway analysis is necessary to confirm the activation of thermogenesis, as well as adipocyte browning. This would provide deeper insights into the molecular mechanisms driving the observed effects and potentially uncover new targets for therapeutic intervention.* Additionally, while *Prdm16* knockdown has provided valuable insights into its role in adipocyte browning, alternative gene silencing techniques, such as CRISPR-based approaches, may offer more precise manipulation of gene expression, minimising the risk of off-target effects and improving the overall understanding of *Prdm16*'s function in thermogenesis.

## 5 Conclusions

In conclusion, this study provides an initial understanding of how bilimbi fractions, phytol, and squalene influence gene and protein expressions in relation to thermogenic adipocyte differentiation. The data suggest that these natural compounds hold potential as therapeutic agents for obesity and related metabolic disorders. However, this research is exploratory, focusing on identifying gene expression changes and key protein markers rather than delving into detailed mechanistic pathways. Future studies will be necessary to investigate the specific signalling pathways, receptors, and downstream molecules activated by these phytocompounds, as well as to fully understand their effects in vivo.

## CRediT authorship contribution statement

**Farah Md Fauzi:** Writing – original draft, Visualization, Methodology, Investigation, Formal analysis, Data curation, Conceptualization. **Mohamad Faiz Hamzah:** Validation, Resources, Methodology, Investigation, Formal analysis. **Muhd Zulkarnain Mahmud:** Visualization, Resources, Investigation, Data curation. **Azimah Amanah:** Validation, Software, Formal analysis. **Mohd Hasnan Mohd Noor:** Resources. **Zafarina Zainuddin:** Writing – review & editing, Supervision, Project administration, Conceptualization. **Wai Kwan Lau:** Writing – review & editing, Visualization, Validation, Supervision, Resources, Project administration, Methodology, Funding acquisition, Conceptualization.

## Data availability statement

Data associated with this study has been include included in the article and its supplementary material. Any additional data referenced in this article can also be found within the main text.

## Declaration of generative AI and AI-assisted technologies in the writing process

The author used OpenAI, CPT-4.0 to enhance language and readability during the writing process. The author reviewed and revised the content as necessary and takes full responsibility for the publication's content.

## Funding information

This work was supported by the (FRGS/1/2019/STG05/MOSTI//4).

## Declaration of competing interest

The authors declare that they have no known competing financial interests or personal relationships that could have appeared to influence the work reported in this paper.
