# Supplementary material for: Phytol and bilimbi phytocompounds induce thermogenic adipocyte differentiation: An in vitro study on potential anti-obesity effects
Source: Heliyon. 2024 Nov 22;10(23):e40518. doi: 10.1016/j.heliyon.2024.e40518 (PMC11652845; doi:10.1016/j.heliyon.2024.e40518)
Supplement: Multimedia component 1 [file mmc1.docx]

**Supplementary Files**


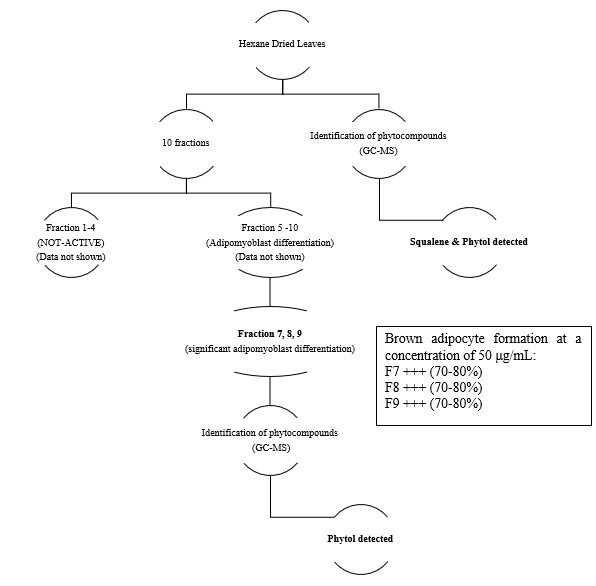


Figure S1: Overview of fraction selection process.

Table S1: Primers used for qRT-PCR analysis. The table includes information about the forward and reverse primer sequences, their 5’ to 3’ orientation and the expected product size in base pairs (bp) for each targeted gene.

| Primers | Forward (5’-3’) | Reverse (5’-3’) | Product size (bp) |
| --- | --- | --- | --- |
| *β-actin* | CTG AAT GGC CCA GGT CTG A | CCC TCC CAG GGA GAC CAA | 80 |
| *Ucp1* | CGT CCC CTG CCA TTT ACT GT | GGT ACG CTT GGG TAC TGT CC | 123 |
| *Cebpα* | AAT GGC AGT GTG CAC GTC TA | CCC CAG CCG TTA GTG AAG AG | 109 |
| *Cebpβ* | GGA TCA AAC GTG GCT GAG CG | GAT TAC TCA GGG CCC GGC TG | 79 |
| *Pgc1α* | TGC CTT CAG TTC ACT CTC AGT | CAA TCC ACT CTG ACA CACAGC | 95 |
| *Prdm16* | TGT CAA GGT GTT CAC GGA CC | AGG ATG TGG CGA AGG TCT TG | 110 |


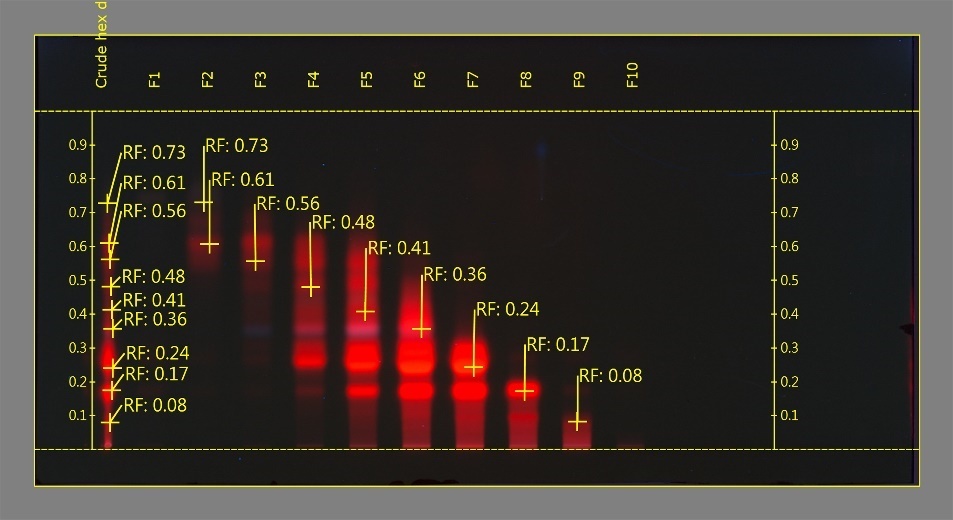


(A)


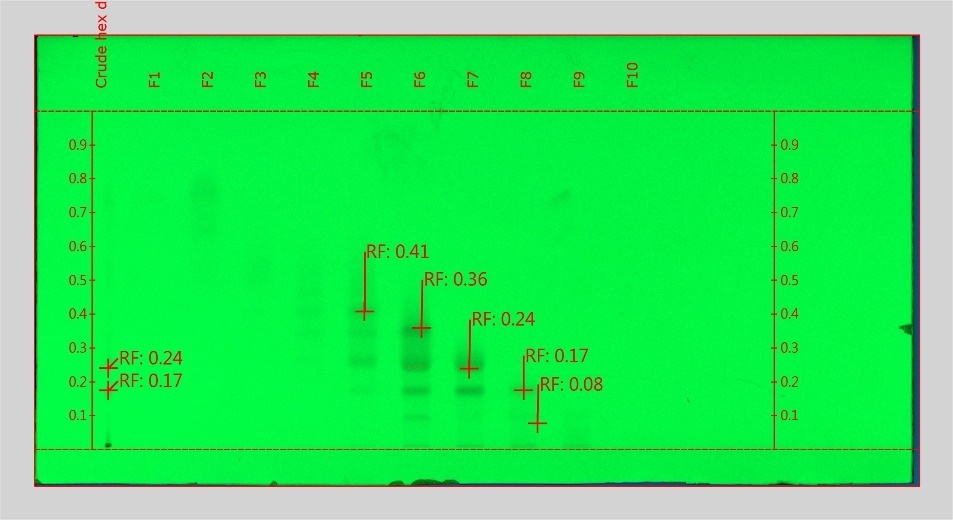


(B)


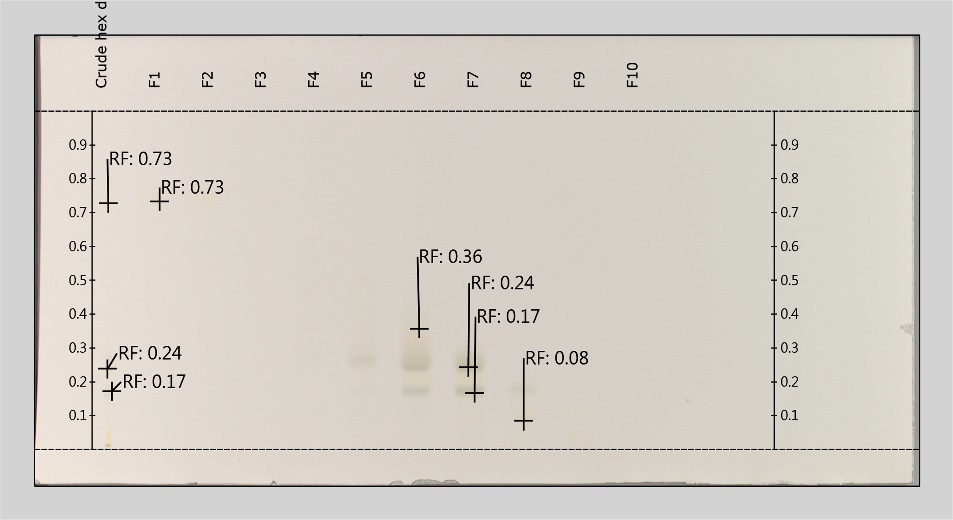


(C)

Figure S2: Thin layer chromatography (TLC) plates displaying the separation of compounds in Bilimbi Fractions (F1-F10), overlaid with a standard Bilimbi crude sample using the mobile phase hexane: ethyl acetate (80:20, v/v). (A) The TLC plate image was viewed under UV 366 nm (long wavelength). (B) The TLC plate image was viewed under UV 254 nm (short wavelength). (C) The TLC plate image was viewed under white light illumination. The distance travelled by each compound above the origin is quantified as the retention factor (R_f_), indicating the relative mobility of each compound in

Table S2: Major chemical components identified in hexane bilimbi leaves extract using GC-MS.RT represents the retention time, which signifies the time taken for the compound to elute from the GC column, along with the common or synthetic name of the identified compounds in the extract and their respective molecular formulas and weights. Area (%) denotes the relative area percentage of the compound’s peak in the chromatogram. M/Z indicates the mass-to-charge ratio used for compound identification. Score (%) indicates the reliability percentage of the identification.

| RT | Name | Molecular Formula | Molecular Weight | Area% | M/Z | Score (%) |
| --- | --- | --- | --- | --- | --- | --- |
| 3.140 | Benzene, 1,4-diethyl- | C_10_H_14_ | 134 | 0.35 | 119.1 | 97.9 |
| 3.675 | Maltol | C_6_H_6_O_3_ | 126 | 0.36 | 126 | 95.3 |
| 5.913 | Phosphonic acid | C_36_H_75_O_3_P | 586 | 0.03 | 69.1 | 79.7 |
| 6.018 | 1-Octadecanol | C_18_H_38_O | 270 | 0.04 | 69.1 | 82.8 |
| 6.980 | Cyclohexadecane | C_16_H_32_ | 224 | 0.01 | 55 | 84.6 |
| 7.563 | (E)-4-(2,6,6-trimethyl-2-cyclohexen-1-yl)-3-buten-2-one | C_13_H_20_O | 192 | 0.02 | 121 | 80.9 |
| 8.000 | Oxalic acid, butyl 6-ethyl oct-3-yl ester | C_16_H_30_O_4_ | 286 | 0.03 | 41.1 | 66.5 |
| 8.659 | 1-Dodecanol, 3,7,11-trimethyl- | C_15_H_32_O | 228 | 0.12 | 69.1 | 75.1 |
| 8.857 | Disulfide, di-tert-dodecyl | C_24_H_50_S_2_ | 402 | 0.11 | 71.1 | 76.6 |
| 8.933 | 2(4H)-Benzofuranone, 5,6,7,7a-tetrahydro-4,4,7a-trimethyl-, (R)- | C_11_H_16_O_2_ | 180 | 0.23 | 111 | 96.3 |
| 9.090 | Octanoic acid | C_18_H_16_O_2_ | 144 | 0.07 | 73 | 57.1 |
| 9.533 | Octane, 2,4,6-trimethyl- | C_11_H_24_ | 156 | 0.02 | 57 | 74.4 |
| 10.069 | 3-Hydroxy-.alpha.-ionene | C_13_H_20_O_2_ | 208 | 0.08 | 109 | 71.1 |
| 10.640 | 3-Buten-2-one, 4-(4-hydroxy-2,2,6-trimethyl-7-oxabicyclo[4.1.0]hept-1-yl)- | C_13_H_20_O_3_ | 224 | 0.12 | 123.1 | 84.6 |
| 10.821 | Tricosane | C_23_H_48_ | 324 | 0.03 | 71.1 | 71.8 |
| 11.830 | 3,6-Dimethyl-5-hepten-1-ol acetate | C_11_H_20_O_2_ | 184 | 0.08 | 124 | 63.8 |
| 12.389 | Neophytadiene | C_20_H_38_ | 278 | 0.84 | 68.1 | 96.2 |
| 12.453 | 2-Pentadecanone, 6,10,14-trimethyl- | C_18_H_36_O | 268 | 1.20 | 58 | 88.4 |
| 12.733 | 2-Hexadecen-1-ol, 3,7,11,15-tetramethyl-, [R-[R*, R*-(E)]]- | C_20_H_40_O | 296 | 0.15 | 81.1 | 83 |
| 13.228 | 3,7,11-trimethyldodecane-2,6,10-trien-1-yl palmitate | C_31_H_56_O_2_ | 460 | 0.17 | 69.1 | 80.3 |
| 13.642 | Farnesyl Acetone C | C_18_H_30_O | 262 | 0.21 | 69.1 | 83.5 |
| 13.689 | Hexadecanoic acid, methyl ester | C_17_H_34_O_2_ | 270 | 0.25 | 74 | 77.6 |
| 14.324 | Hexadecanoic acid | C_16_H_32_O_2_ | 256 | 0.24 | 73 | 81.9 |
| 17.786 | Phytol | C_20_H_40_O | 296 | 10.32 | 71.1 | 98.5 |
| 18.404 | 9-Octadecenoic acid (Z)- | C_18_H_34_O_2_ | 282 | 1.01 | 67.1 | 73.7 |
| 18.719 | 9,12-Octadecadienoic acid (Z,Z)- | C_18_H_32_O_2_ | 280 | 0.30 | 55.1 | 70.4 |
| 20.135 | 3,7,11,15-Tetramethylhexadec-2-en-1-yl acetate | C_22_H_42_O_2_ | 338 | 0.83 | 123.1 | 92.5 |
| 20.928 | 9-Octadecene, 1,1-dimethoxy-, (Z)- | C_20_H_40_O_2_ | 312 | 0.11 | 57.1 | 63.5 |
| 21.744 | 2-Bromotetradecane | C_14_H_29_Br | 276 | 0.75 | 57.1 | 60.4 |
| 22.193 | 2,6,10,14-Hexadeca-Tetraen-1-ol, 3,7,11,15-tetramethyl-, acetate, (E, E, E)- | C_22_H_36_O_2_ | 332 | 0.24 | 69.1 | 70.4 |
| 22.607 | Thiophene, 3-methyl-2-pentadecyl- | C_20_H_36_S | 308 | 0.26 | 111 | 65.6 |
| 22.805 | 4,8,12,16-Tetramethylheptadecan-4-olide | C_21_H_40_O_2_ | 324 | 0.38 | 99 | 83.6 |
| 23.399 | 5,9,13-Pentadecatrien-2-one, 6,10,14-trimethyl- | C_18_H_30_O | 262 | 0.26 | 69.1 | 75 |
| 25.422 | 7,7-Di-ethylheptadecane | C_21_H_44_ | 296 | 0.16 | 57.1 | 62.4 |
| 26.285 | Bis(2-ethylhexyl) phthalate | C_24_H_38_O_4_ | 390 | 0.77 | 149 | 92.5 |
| 27.182 | Octadecanoic acid, phenyl ester | C_24_H_40_O_2_ | 360 | 0.08 | 57 | 53.2 |
| 29.275 | Docosane | C_22_H_46_ | 310 | 0.13 | 57.1 | 55.6 |
| 30.353 | Solanesol | C_45_H_74_O | 630 | 0.17 | 69.1 | 64.1 |
| 32.440 | Pregn-16-en-20-one, 3-(acetyloxy)-, (3.alpha.)- | C_23_H_34_O_3_ | 358 | 0.22 | 69.1 | 54.0 |
| 32.842 | Squalene | C_30_H_50_ | 410 | 63.51 | 69.1 | 96.9 |
| 33.535 | .alpha.-Tocospiro A | C_29_H_50_O_4_ | 462 | 0.60 | 419.3 | 78.5 |
| 34.287 | .alpha.-Tocospiro B | C_29_H_50_O_4_ | 462 | 0.30 | 419.3 | 66.7 |
| 35.156 | Celidoniol, Deoxy- | C_29_H_60_ | 408 | 3.35 | 57.1 | 90.7 |
| 36.479 | 1,6,10,14,18,22-Tetracosahexaen-3-ol, 2,6,10,15,19,23-hexamethyl-, (all-E)-(.+/-.)- | C_30_H_50_O | 426 | 2.40 | 69.1 | 85.7 |
| 36.718 | .delta.-Tocopherol | C_27_H_46_O_2_ | 402 | 4.32 | 402.4 | 96.4 |
| 37.202 | trans-Geranylgeraniol | C_20_H_34_O | 290 | 0.58 | 69.1 | 71.4 |
| 37.423 | Solanesol | C_45_H_74_O | 630 | 0.63 | 69.1 | 71.3 |

Table S3: Major chemical components identified in fractions (7, 8, and 9) of hexane bilimbi leaves extract using GC-MS. RT represents the retention time, indicating the time taken for compound elution from the GC column. It also includes the common or synthetic name of the compounds identified in the extract, along with their respective molecular formulas and weights. Area (%) denotes the relative area percentage of the compound’s peak in the chromatogram. M/Z indicates the mass-to-charge ratio used for compound identification. Score (%) represents the reliability percentage of the identification.

| **RT** | **Name** | **Molecular Formula** | **Molecular Weight** | **Area %** | **M/Z** | **Score (%)** |
| --- | --- | --- | --- | --- | --- | --- |
| **Fraction 7** | | | | | | |
| 3.151 | Benzene, 1,4-diethyl- | C_10_H_14_ | 134 | 0.239573 | 119 | 91.2 |
| 10.687 | 3-Buten-2-one, 4-(5-hydroxy-2, 6, 6-trimethyl-1-cyclohexen-1-yl) | C_13_H_12_O_2_ | 208 | 0.164 | 193.1 | 72.4 |
| 11.614 | 6-Hydroxy-4,4,7a-trimethyl-5,6,7,7a-tetrahydrobenzofuran-2(4H)-one | C_11_H_16_O_3_ | 196 | 0.390179 | 111 | 86.6 |
| 12.377 | Neophytadiene | C_20_H_38_ | 278 | 3.911934 | 68.1 | 96.2 |
| 13.013 | 3,7,11,15-Tetramethyl-2-hexadecen-1-ol | C_20_H_40_O | 296 | 1.100033 | 81 | 86.2 |
| 13.648 | Farnesyl Acetone C | C_18_H_30_O | 262 | 0.213 | 69.1 | 71.2 |
| 17.781 | Phytol | C_20_H_40_O | 296 | 12.66445 | 71.1 | 97.4 |
| 20.922 | .beta.-Amyrin | C_30_H_50_O | 426 | 26.90275 | 218.2 | 92.9 |
| 26.011 | 1-Hexacosanol | C_26_H_54_O | 382 | 19.11634 | 97 | 86.3 |
| 26.273 | Phthalic acid, di(2-propylpentyl) ester | C_24_H_38_O_4_ | 390 | 4.972 | 149.0 | 77.9 |
| 27.853 | 9,19-Cyclolanost-24-en-3-ol, (3-beta.)- | C_30_H_50_O | 426 | 5.498 | 69.0 | 75.8 |
| 36.432 | Solasenol | C_45_H_74_O | 630 | 1.125 | 69.1 | 71.8 |
| 36.695 | .delta.-Tocopherol | C_27_H_46_O_2_ | 402 | 2.528389 | 402.4 | 87.9 |
| **Fraction 8** | | | | | | |
| 3.1512 | Benzene, 1,4-diethyl- | C_10_H_14_ | 134 | 0.40001 | 119.1 | 95.6 |
| 8.933 | 2(4H)-Benzofuranone, 5,6,7,7a-tetrahydro-4,4,7a-trimethyl-, (R)- | C_11_H_16_O_2_ | 180 | 0.329944 | 111.1 | 87.4 |
| 10.069 | 3-Cyclohexene-1-carboxaldehyde, 1,3,4-trimethyl- | C_10_H_16_O | 152 | 0.376866 | 109.1 | 73.7 |
| 10.384 | Spiro[3.5]nonan-1-ol, 1,5,5,9-tetramethyl-, [4.alpha.(S*),9.beta.]- | C_13_H_24_O | 196 | 0.345515 | 181.1 | 71.0 |
| 10.641 | 3-Buten-2-one, 4-(4-hydroxy-2,2,6-trimethyl-7-oxabicyclo[4.1.0]hept-1-yl)- | C_13_H_20_O_3_ | 224 | 1.262744 | 123.1 | 89.0 |
| 10.687 | 3-Hydroxy-7,8-dihydro-.beta.-ionol | C_13_H_20_O_2_ | 208 | 0.624288 | 193.1 | 70.0 |
| 10.856 | 2-Cyclohexen-1-one, 4-(3-hydroxybutyl)-3,5,5-trimethyl- | C_13_H_22_O_2_ | 210 | 0.36553 | 135.1 | 89.2 |
| 11.614 | 6-Hydroxy-4,4,7a-trimethyl-5,6,7,7a-tetrahydrobenzofuran-2(4H)-one | C_11_H_16_O_3_ | 196 | 2.868727 | 111 | 96.5 |
| 12.383 | Neophytadiene | C_20_H_38_ | 278 | 6.133766 | 68.1 | 95.6 |
| 12.727 | 3,7,11,15-Tetramethyl-2-hexadecen-1-ol | C_20_H_40_O | 296 | 0.463731 | 82.1 | 82.6 |
| 13.019 | 3,7,11,15-Tetramethyl-2-hexadecen-1-ol | C_20_H_40_O | 296 | 1.585884 | 81.1 | 87.3 |
| 15.146 | gamma-Sitosterol | C_29_H_50_O | 414 | 1.515095 | 414.4 | 73.7 |
| 17.786 | Phytol | C_20_H_40_O | 296 | 13.581 | 71.1 | 97.1 |
| 20.934 | beta-Amyrin | C_30_H_50_O | 426 | 10.363 | 218.2 | 80.0 |
| 25.993 | 1-Hexacosanol | C_26_H_54_O | 382 | 19.403 | 97.1 | 77.2 |
| 26.273 | Bis(2-ethylhexyl) phthalate | C_24_H_38_O_4_ | 390 | 8.126355 | 149 | 91.0 |
| 32.457 | (3methyl,24R)-ergost-5-en-3-ol | C_28_H_­48_O | 400 | 4.520 | 400.4 | 78.6 |
| 34.969 | Stigmasterol | C_29_H­_48_O | 412 | 5.288 | 55.0 | 79.4 |
| **Fraction 9** | | | | | | |
| 3.145 | Benzene, 1,4-diethyl- | C_10_H_14_ | 134 | 8.13231 | 119.1 | 93.2 |
| 3.518 | 4,6-Dimethyl-3-nonanone | C_11_H_22_O | 170 | 1.841589 | 57.1 | 79.5 |
| 10.309 | 3-Acetyl-2-methyl-9b-(1-naphthyl)-3aH-benzo[e]cyclohexa[1,2-b] furan | C_25_H_22_O_2_ | 354 | 0.949077 | 355.1 | 79.9 |
| 10.682 | 3-Buten-2-one, 4-(5-hydroxy-2,6,6-trimethyl-1-cyclohexen-1-yl)-, [S-(E)]- | C_13_H_20_O_2_ | 208 | 0.965028 | 193 | 62.8 |
| 11.602 | 2(4H)-Benzofuranone, 5,6,7,7a-tetrahydro-6-hydroxy-4,4,7a-trimethyl-, (6S-cis)- | C_11_H_16_O_3_ | 196 | 7.241251 | 111 | 79.1 |
| 12.191 | N-Bis(trimethylsilyl)methyl-2-benzylmethyl-3-(2-oxo-4,5-diphenyloxazolidin-3-yl)-1-azacyclobutan-4-one | C_33_H_42_N_2_O_3_Si_2_ | 570 | 1.25266 | 281.1 | 70.5 |
| 12.378 | Neophytadiene | C_20_H_38_ | 278 | 12.20493 | 68.1 | 75.7 |
| 13.001 | (Z,E)-10,12-Pentadecadienol | C_15_H_28_O | 224 | 1.375244 | 67 | 71.5 |
| 17.775 | Phytol | C_20_H_40_O | 296 | 2.866 | 71 | 74.1 |
| 26.267 | Phthalic acid, di(2-propylpentyl) ester | C_24_H_38_O_4_ | 390 | 22.6896 | 149 | 82.5 |

(A)

(B)

Figure S3: GC-MS chromatogram profiles showing standard solutions of (A) phytol and (B) squalene

Figure S4: Effect of transfection efficiency on the expression of mRNA *PRDM16* in C2C12 myoblasts.

The siRNA control experiments consist of the untransfected group, the mock-transfected group (the cells transfected with transfection reagent only), the negative, positive control siRNA, and *PRDM16* siRNA. The concentration of siRNAs used was 10 nM. Relative mRNA expression (measured by qRT-PCR) of *Prdm16* was determined by normalization to β-actin and compared to the untransfected group, which was assigned the value of 1. Data represent the mean of means ± SEM (n=3). * indicates statistical significance for ANOVA followed by Dunnett’s t-test multiple comparison test between siRNA control and untransfected groups for p ≤ 0.05.

The expression of *Prdm16* in siRNA *Prdm16*-transfected cells was five times lower than that in other

siRNA controls, achieving an 80% knockdown. The expression levels of *Prdm16* in scrambled siRNA controls, negative siRNA controls, and mock-transfected cells were comparable to those in untransfected cells, confirming the effectiveness and specificity of the gene knockdown

Table S4: Quantification of UCP1 protein levels, normalised to beta actin, represented as fold expression relative to controls for both non-transfected and transfected C2C12 myoblasts. Statistical significance was determined by one-way ANOVA followed by Dunnett’s post hoc test, with p ≤ 0.05 considered statistically significant (n=3).

| Cell type | DMSO | ROSI | F7 | F8 | F9 | SQ | PHY |
| --- | --- | --- | --- | --- | --- | --- | --- |
| Non-transfected C2C12 | 0.21 ±0.05 | 0.61 ± 0.10 | 0.65 ±0.15 | 0.70 ± 0.14 | 0.68 ± 0.12 | 0.83 ± 0.08 | 0.99 ± 0.20 |
| siRNA-*Prdm16* transfected C2C12 | 0.5 ±0.14 | 1.31±0.17 | 0.70±0.12 | 0.86±0.16 | 1.03 ± 0.24 | 1.17 ± 0.15 | 1.30 ± 0.18 |


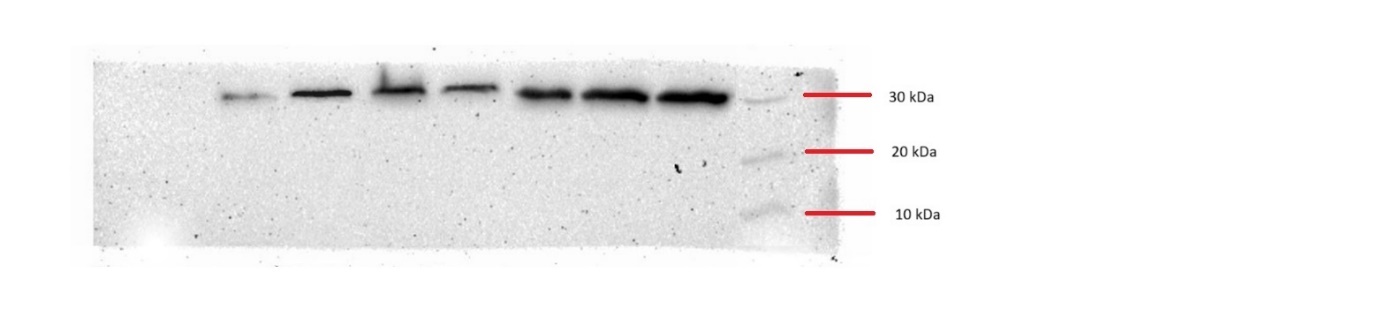


Figure S5: Representative western blot image of the UCP1 protein levels in non-transfected C2C12 myoblasts. UCP1, a 30 kDa protein, was detected using a primary antibody against UCP1, followed by an HRP-conjugated secondary antibody.


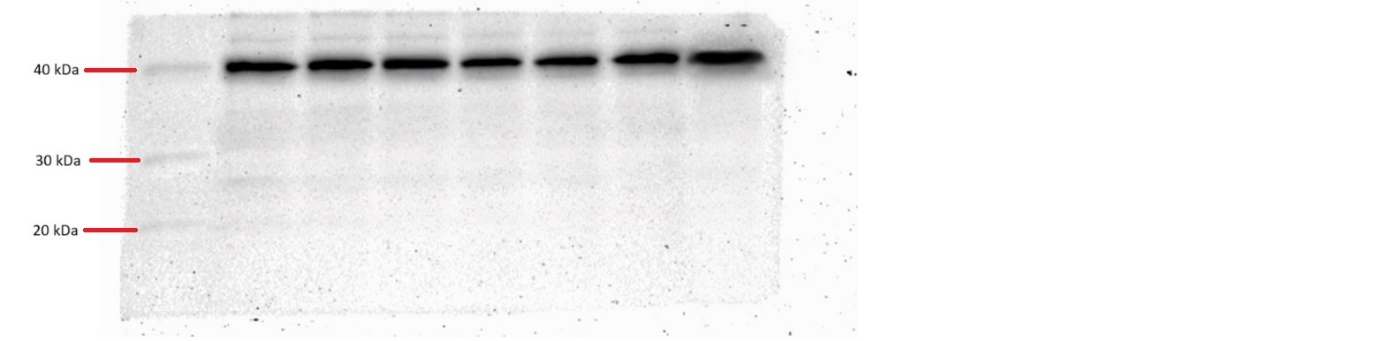


Figure S6: Representative western blot image of β-actin protein levels in non-transfected C2C12 myoblasts. β-actin (42 kDa) was used as the loading control.


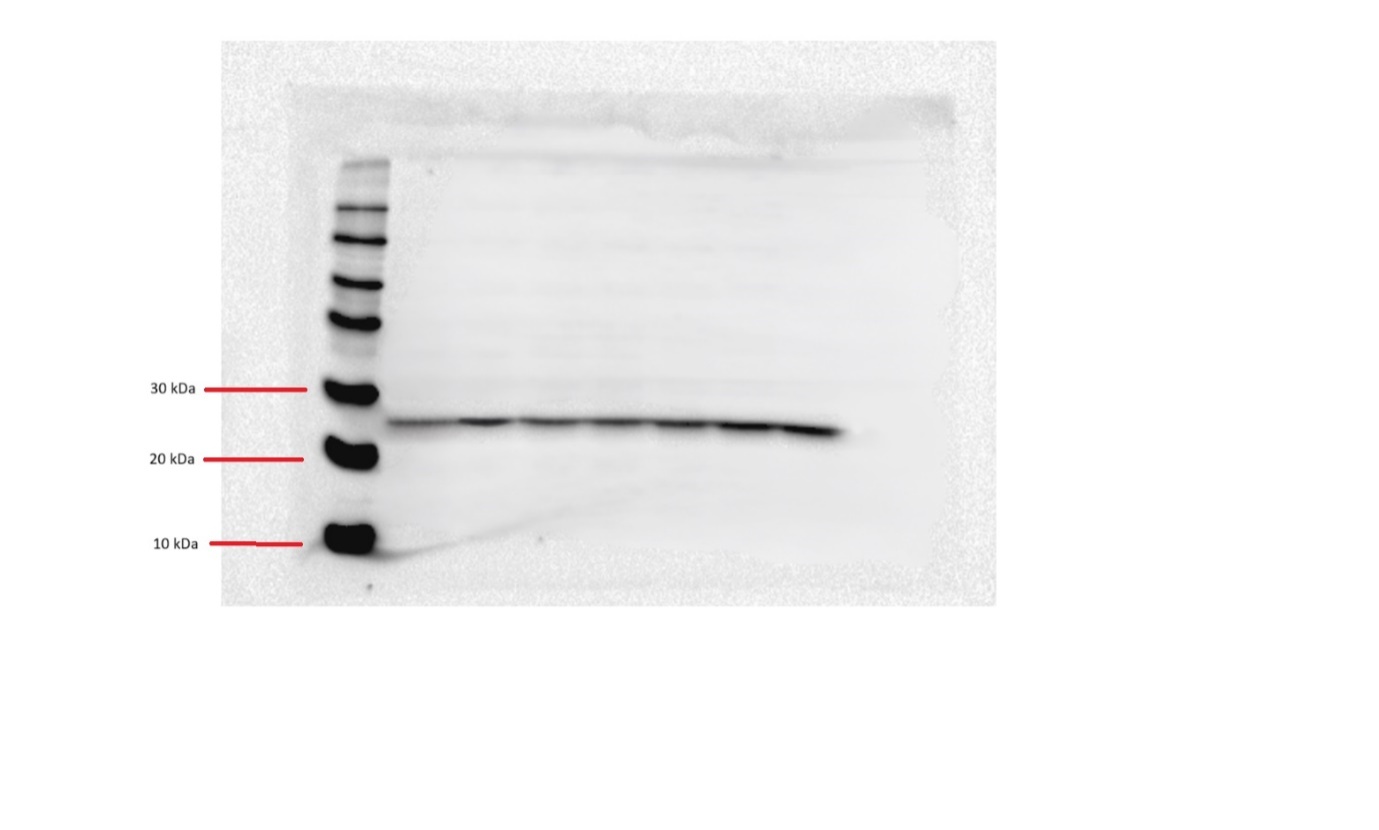


Figure S7: Representative western blot image of UCP1 protein in siRNA-*Prdm16* silenced C2C12 myoblasts. UCP1, a 30 kDa protein, was detected using a primary antibody against UCP1, followed by an HRP-conjugated secondary antibody.


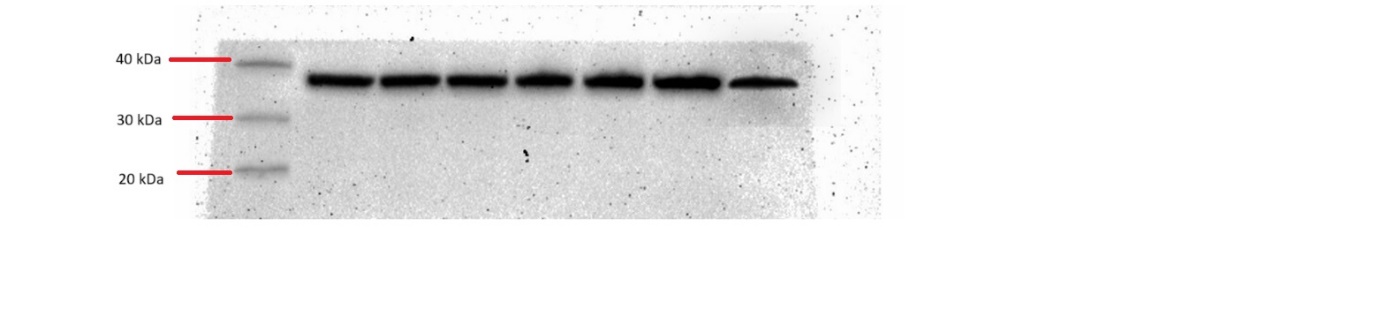


Figure S8: Representative western blot image of β-actin protein in siRNA-*Prdm16* silenced C2C12 myoblasts. β-actin (42 kDa) was used as the loading control.
